# X-ray powder diffraction in education. Part I. Bragg peak profiles

**DOI:** 10.1107/S1600576721009183

**Published:** 2021-10-27

**Authors:** Robert Dinnebier, Paolo Scardi

**Affiliations:** a Max Planck Institute for Solid State Research, Heisenbergstrasse 1, Stuttgart, 70569, Germany; b Department of Civil, Environmental and Mechanical Engineering, University of Trento, via Mesiano 77, Trento, 38123, Italy

**Keywords:** powder diffraction, line profile analysis, peak shape function, microstructure, *Mathematica*

## Abstract

A collection of scholarly scripts dealing with the mathematics and physics of peak profile functions in X-ray powder diffraction has been written using the Wolfram language in *Mathematica*. Common distribution functions, the concept of convolution in real and Fourier space, instrumental aberrations, and microstructural effects are visualized in an interactive manner and explained in detail. This paper is the first part of a series dealing with the mathematical description of powder diffraction patterns for teaching and education purposes.

## Abstract 2

Peak profile functions in powder diffraction are presented with the support of *Mathematica* scripts, easily usable by interested readers, to explore the effect of different instrumental and microstructural parameters. Subsequent articles will illustrate the most common aberrations to the measured patterns and then total scattering methods.

## 1. Introduction

X-ray powder diffraction (XRPD), an established technique for the study of crystal structures, finds increasing application in qualitative and quantitative phase analysis and the study of microstructure for micro- and nanocrystalline materials.

For the study of crystalline materials, a powder pattern can be divided into Bragg reflections and background, where the latter among other contributions contains diffuse scattering from the sample. Bragg reflections are characterized by their position, intensity, breadth and shape, each containing a wealth of information (Dinnebier & Billinge, 2008 ▸; Dinnebier *et al.*, 2018 ▸).

Computer programs for fitting peak profiles in powder diffraction perform either single peak fits or whole powder profile fitting with or without a structural model. Many programs have in common the ability to work like a black box for the user. It is frequently the case that some parameters are little affected by the nonlinear least-squares procedure, while others change wildly. In particular, among non-experts, a lot of confusion exists regarding the meaning and significance of fixed or refined parameters, their contribution to the Bragg peaks, the quality of the fit, the range of convergence, precision and accuracy, meaning and reliability of standard deviations *etc*. In addition, many functions describing a physical effect show discontinuities, and the refined parameters are typically restricted by physical boundaries. Understanding the algorithms is necessary for correct use of the software and assessment of the reliability of results.

In this series of papers dealing with the visualization of mathematical functions used to describe a powder pattern, we present a collection of user-friendly, interactive and freely distributable *Mathematica* (Wolfram Research, https://mathworld.wolfram.com/) teaching scripts. All scripts have been written in Wolfram *Mathematica* version 12.1.1.0 and are constantly updated. They are freely available at the *TOPAS* Wiki web site (http://topas.dur.ac.uk/topaswiki). Non-subscribers of *Mathematica* can run the scripts using the freely available *Wolfram Player* at https://www.wolfram.com/player/. Bugs and problems should be reported to r.dinnebier@fkf.mpg.de. In particular, the so-called ‘Manipulate’ option of *Mathematica* is extensively used to visualize the impact of parameters in an interactive manner. When possible, parameters from real-life examples are used as the default. The idea is ‘learn by doing’, to gain intuition for what a mathematical model does to the diffraction peaks in a powder pattern and what the limitations of the said model are, since every model is an oversimplification of the underlying physics.

All parameters of the scripts are taken from the two well established programs *TOPAS* (Coelho, 2018 ▸) and *WPPM* (Scardi & Leoni, 2001 ▸, 2002 ▸; Scardi *et al.*, 2018 ▸; Scardi, 2020 ▸).

At the moment, the following three parts of this series are envisaged: part I, the peak profile of a powder pattern (this paper); part II, common correction functions in powder diffraction; part III, total scattering.

In this first paper, the various contributions to the peak profile in a powder diffraction pattern are described in terms of physical models and the effect they have on the peak shape. In general, the profile of a Bragg peak has contributions from the diffractometer and the microstructure of the sample. In the past few decades, a variety of sophisticated techniques and computer programs for analysing the peak shape of a powder pattern have been developed (Dinnebier & Billinge, 2008 ▸).

In general, the profile 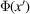 of a Bragg reflection centred at the peak position 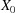 can be approximated by mathematical convolution (denoted by the symbol 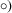 of contributions from the instrument, called the instrumental resolution function (IRF), and from the microstructure (MS) of the sample (Klug & Alexander, 1974 ▸):

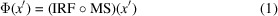

with

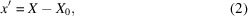

where 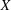 is the observed peak position on the scale in which the data are recorded or analysed. The profile function is therefore described relative to the peak centre 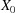 .

Since the majority of powder patterns are directly measured as a function of the scattering angle 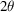 in degrees, we will use 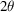 as an independent variable for our profile functions throughout the text. Note that many of the aberrations to be discussed in part II are specifically 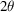 dependent. The conversion to reciprocal space in nm^−1^ is given by the Bragg equation:

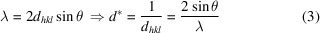

with the wavelength 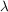 in nm and the distance between lattice planes 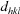 with indices *hkl* in nm. Other scales besides 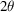 , *d* and 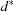 include *Q* (= 2π 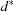 ), time of flight (TOF) and energy (*E*). The conversion factor

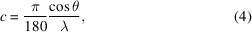

which follows from the differential of the Bragg equation, is used to change space from 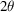 in degrees to 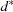 in nm^−1^. [The factor π/180 is used to convert an angular value given in degrees (*e.g.* the breadth of a reflection) to radians.] The Fourier transformation (FT) of a function in 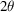 space in degrees leads to a function with reciprocal angle and units of degree^−1^ that we will use for illustrative purposes only. The FT of a function in 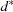 space and units of nm^−1^ leads to a function in nm on a length scale in real space.

The IRF itself can be considered a convolution of contributions coming from the finite width of the X-ray source (X-ray tube or synchrotron), called the emission profile, and a series of horizontal and vertical instrumental aberrations due to the diffractometer. In the most popular configuration, these include the angular acceptance function of the Soller slit(s) controlling the axial beam divergence, the angular acceptance function of the plug-in slit controlling the equatorial beam divergence, the angular acceptance function of the receiving slit *etc*. For linear position-sensitive detector (PSD) systems, the receiving slit aberration is replaced by functions describing the defocusing due to asymmetric diffraction, the parallax error and the point spread function of the detector:

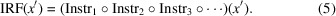

The same principle holds for the MS contribution of the sample, which can be viewed as a convolution of contributions from effects like the size of coherently scattering domains, isotropic and/or anisotropic microstrain, faulting *etc*.:

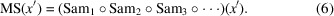

Equations (5) and (6) implicitly assume that all components of the convolution are independent, which is a common approximation but not exact for MS parameters.

To describe the peak shape in a powder diffraction pattern, two approaches are common nowadays: (i) Ideally, all contributions and their mathematical description are known. The resulting peak shape can thus be calculated from first principles. For building the IRF, the fundamental parameters (FP) approach (Cheary & Coelho, 1992 ▸, 1998 ▸) and for the MS the whole powder pattern modelling (WPPM) concept (Scardi & Leoni, 2001 ▸, 2002 ▸) are quite common. (ii) Alternatively, because many contributions to powder diffraction peaks have a nearly Gaussian or Lorentzian shape, the Voigt function, which is a convolution of Gaussian and Lorentzian components, or more commonly the pseudo-Voigt function for faster computation, is widely used to describe peak profiles in powder diffraction. Importantly, the latter approach is empirical, and all interpretations of MS parameters based on the Voigt profile must be carefully evaluated.

In fact, real instruments are always more complex than any possible FP model. The differences between model and reality can be a source of systematic errors with the risk of ignoring some features of the IRF. As a commonly accepted compromise, the IRF is obtained by fitting a line profile standard with virtually no MS contributions using an empirical Voigt profile, eventually convoluted with some instrumental aberration functions. In contrast, the MS is derived from more physical or at least phenomenological functions with few but meaningful parameters.

## 2. Fourier transformation and convolution

The FT greatly simplifies the convolution process and reveals many important properties of the peak profile, making it a good mathematical procedure to begin with.

The FT of a function 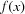 is a complex function defined as

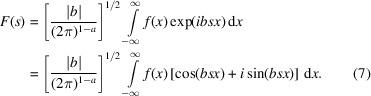




The Fourier integral transforms a function 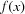 (*x* in real space) by an integral over cosine and sine functions to 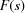 (*s* in reciprocal space = Fourier space). Some common choices for the Fourier parameters {*a*, *b*} which have a big impact on scaling are {0, 1} (modern physics), {1, −1} (pure mathematics, systems engineering), {−1, 1} (classical physics), {0, −2π} (signal processing) and {0, 2π} (crystallography).

The back-transformation is given by

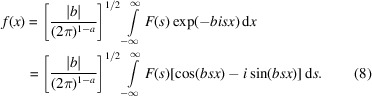




The independent variables, *s* and *x*, are reciprocal to one another, so the exponent of the exponential function is dimensionless. If *x* is in position space, *s* is in ‘Fourier space’ or ‘reciprocal space’. In the following, we will use the crystallographic Fourier parameters {0, 2π} for simplicity:

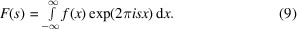

Convolution, or folding, is a basic concept in crystallography that is particularly important in powder diffraction analysis. The process of convolution is one in which the product of two functions 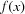 and 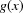 is integrated over all space:

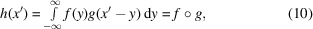

where 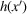 is the convolution product, *y* is the variable of integration in the same domain as 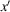 and 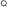 denotes the convolution process. Convolution can be understood as ‘blending’ one function with another, producing a kind of very general ‘moving average’ [see Weisstein (2021 ▸) for a definition and animated examples]. Most functions cannot be convoluted analytically and the convolution integral needs to be calculated numerically.

An alternative method of calculation follows from the convolution theorem of FT. The FT of the convolution function can be calculated by

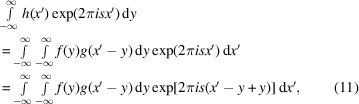

which can be rewritten using the substitution 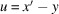 and therefore 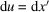 as

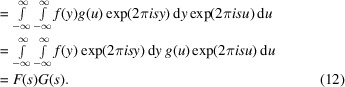




From the last, it follows directly that the FT of the convolution integral is the product of the FTs of all functions participating in the convolution,

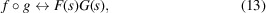

while the back-transformation of a convolution is the product of the back-transformed functions which participate in the convolution:

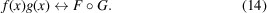

In many cases, an analytic FT of a mathematical function is not possible, requiring the numerical FT of a finite list of *N* function values 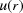 at index *r* (running from 1 to *N*), which are separated by a constant step width 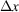 . The corresponding running index in Fourier space is *s* (running from 1 to *N*) and the FT values are separated by a constant step width. This means that some piece of the integral can be discretized by

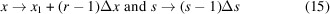

with

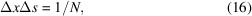

leading to

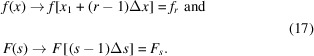




Equation (7) can thus be written in a discretized form as

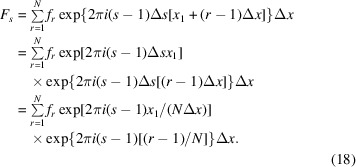




In *Mathematica*, a numerical discrete FT is defined as 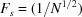 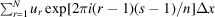 , from which it follows that the 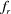 values must be multiplied by a complex phase factor 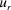 = 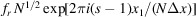 (*Mathematica* stackexchange questions 1714, https://mathematica.stackexchange.com/questions/1714/numerical-fourier-transform-of-a-complicated-function/151179#151179).

## 3. Probability density functions

The shape of powder diffraction peaks is usually described by individual or convoluted probability density functions (PDFs) used to describe statistical processes. In statistics, PDFs are characterized by their moments, which are quantitative measures related to their shape. The zeroth moment is the total probability (which equals one), the first moment is the expected value (mean), the second central moment is the variance, the third standardized moment is the skewness and the fourth standardized moment is the kurtosis. The variance is the expectation of the squared deviation of a random variable from its mean. Skewness is a measure of the asymmetry of the probability distribution of a real-valued random variable about its mean, and kurtosis is a measure of the ‘tailedness’ of the probability distribution of a real-valued random variable (from Wikipedia).

In powder diffraction, the widths of distributions used to describe peak profiles are either the standard deviation (σ), the full width at half-maximum (FWHM) which is the difference between the two values of the independent variable at which the dependent variable is equal to half of its maximum value (height of the peak), the half-width at half-maximum (HWHM) which is FWHM/2, or the integral breadth (β), the last being the width of a rectangle of the same height and area as the peak.

The standard deviation σ or square root of the variance of a distribution described by *f*(*x*) (which is normalized to an area of unity) is given as

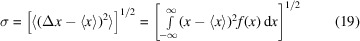

and the average is given as

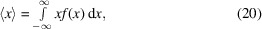

which agrees (in the case of a symmetric unimodal distribution) with the peak maximum *x*_0_. For many common distributions, the variance and therefore the standard deviation are not defined. In these cases, the HWHM, FWHM or β is always used. The last is defined as

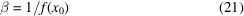

for a function *f*(*x*) that is normalized to an area of unity.

### 3.1. Gaussian distribution

**Figure 1:**
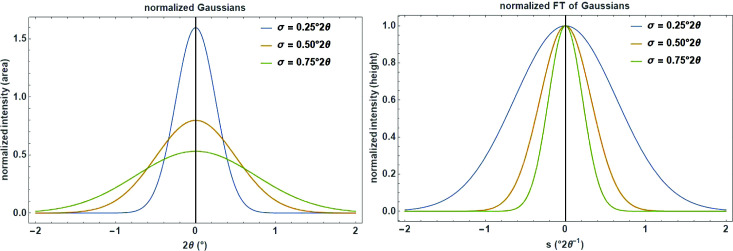
Series of normalized Gaussians (left) and their corresponding Fourier transforms (right) with different standard deviation σ on a 2 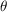 and reciprocal 2 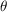 scale.

The Gaussian (or normal) distribution is a very common distribution. Physical quantities expected to be the sum of many independent processes (such as measurement errors) often have distributions that are nearly normal. A Gaussian function normalized to an area of unity centred at a mean or expectation value 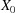 is (Fig. 1 ▸, left)

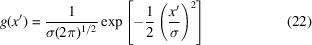

where σ is the standard deviation and 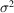 the variance. The pre-factor follows from normalization:

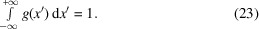

The HWHM of a Gaussian is given by

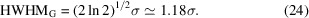

With HWHM_G_ as the width parameter, the definition of the Gaussian changes accordingly to

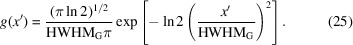

The integral breadth of a Gaussian is given by

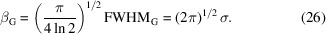

The normalized FT (maximum value is 1) of a Gaussian function is itself a Gaussian function (Fig. 1 ▸, right):

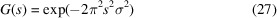

with an FWHM of

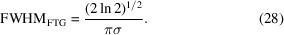

The convolution of a Gaussian (1) with another Gaussian (2) is a Gaussian with the following property:

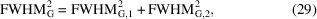

which follows directly from the additivity of variances and also holds for integral breadths.

### 3.2. Lorentz distribution

**Figure 2:**
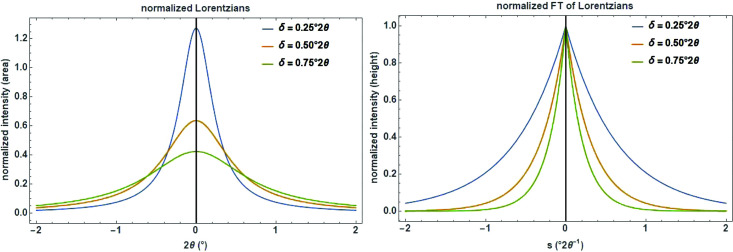
Series of normalized Lorentzians (left) and their corresponding real Fourier transforms (right) with different δ (= HWHM_L_) on a 2 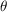 and reciprocal 2 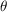 scale.

The Lorentz (or Cauchy) function is another important continuous probability density distribution, which might be attributed, for instance, to the lifetime broadening of the characteristic X-ray emission line. The Lorentz function is used, for example, to describe the emission profile from an X-ray tube and the peak profile in some faulting problems when a random probability exists in the stacking sequence, as well as to approximate crystallite size and strain effects from the sample. Also, in a perfect infinite crystal, Bragg peaks are not δ functions but finite Lorentzians with the FWHM being the Darwin width (Warren, 1990 ▸). The Lorentz distribution normalized to unity is defined as (Fig. 2 ▸, left)

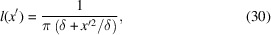

with δ being the Lorentzian HWHM 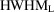 . The Lorentz distribution is an example of a distribution with no mean, no standard deviation, and no variance or higher moments defined. Its mode and median (the value separating the higher half from the lower half of a data sample) are well defined and are both equal to 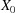 .

The integral breadth of a Lorentzian is given as

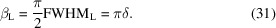

The real part of the FT (normalized to unity) of a Lorentzian is (Fig. 2 ▸, right)

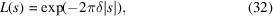

with an FWHM of

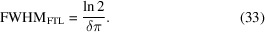

The convolution of a Lorentzian (1) with another Lorentzian (2) is a Lorentzian with the following property:

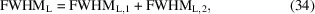

which also holds true for integral breadths.

### 3.3. The Voigt distribution

The Voigt distribution, named after the German physicist Woldemar Voigt (Voigt, 1912 ▸), can be regarded as the convolution of a normalized Gaussian and a normalized Lorentzian:

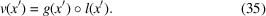

**Figure 3:**
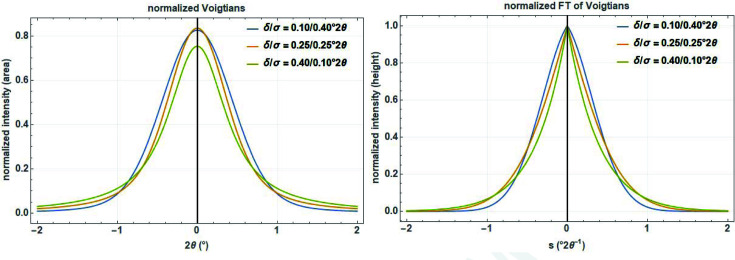
Series of normalized Voigt profiles (left) and their corresponding real Fourier transforms (right) with different Gaussian standard deviation σ and Lorentzian HWHM δ on a 2 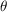 and reciprocal 2 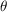 scale.

No analytical solution exists for the convolution integral, but it can be expressed by the real part of the complex error function for which good approximations exist (Fig. 3 ▸, left):

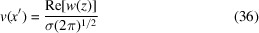

where 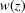 is called the Faddeeva function (also called the Kramp function or relativistic plasma dispersion function) and is a scalable complex conjugated error function. This is given by

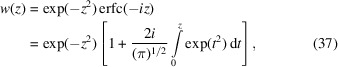

and the argument *z* is

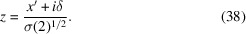

**Figure 4:**
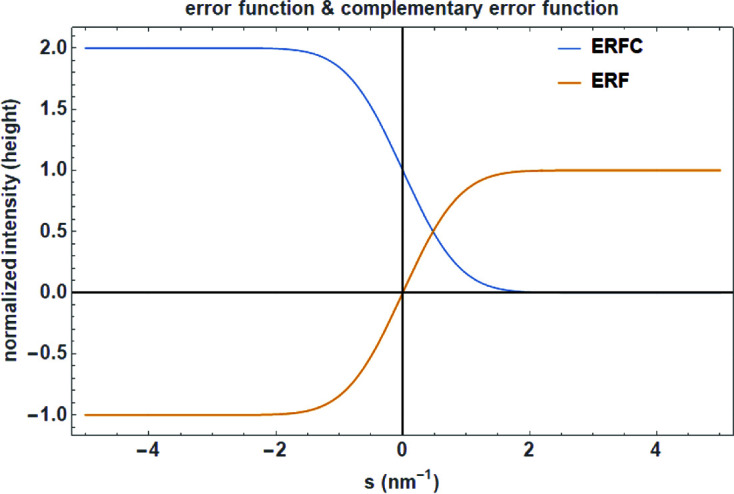
Plot of the error function and the complementary error function given in equations (38) and (39) [from Dinnebier *et al.* (2018 ▸)].

When the results of a series of measurements are described by a normal distribution with standard deviation σ and expected value 0, then

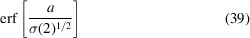

is the probability that the error of a single measurement lies between −*a* and +*a*, for positive *a*. The error function is defined as (Fig. 4 ▸)

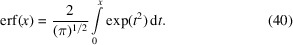

The complementary error function is defined as

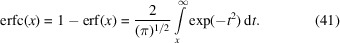

**Figure 5:**
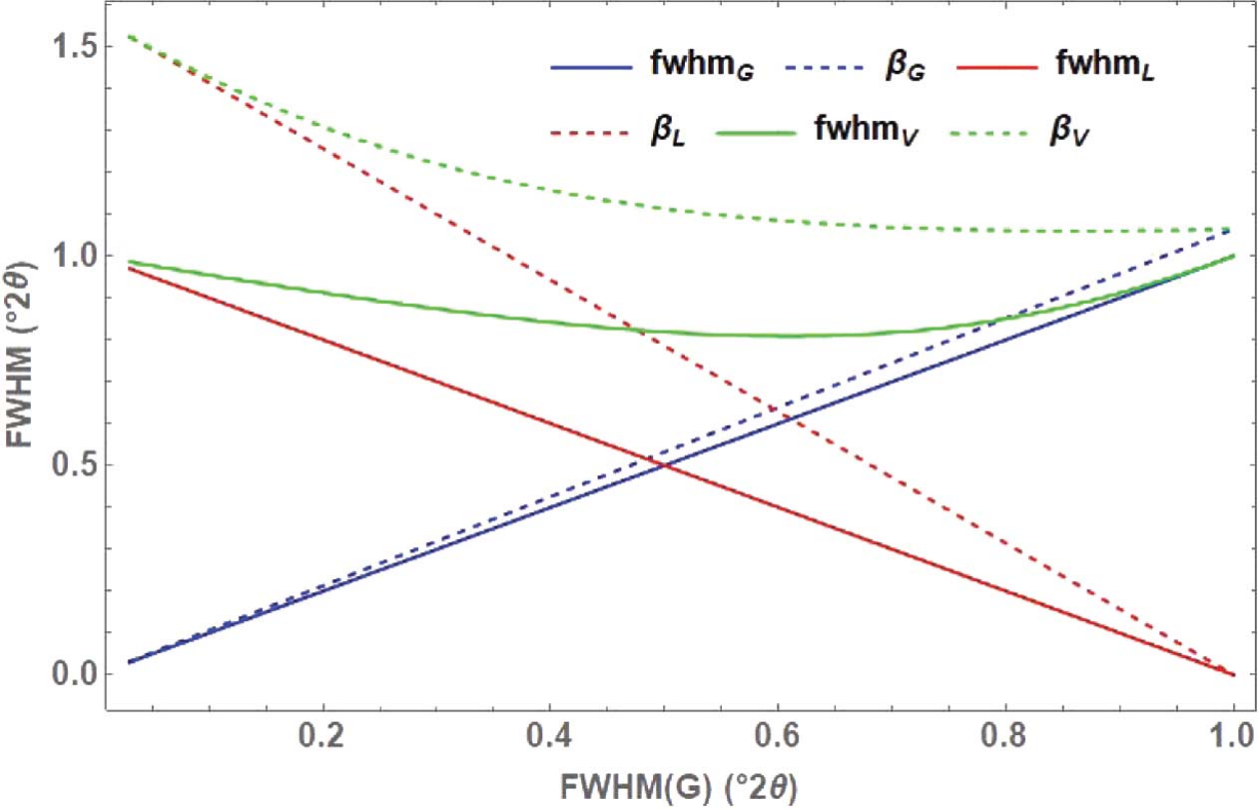
Plot of the FWHM of the TCH pseudo-Voigt function as a function of the FWHMs of the Gaussian and the Lorentzian part (solid lines) and the corresponding integral breadths β (dashed lines).

The FWHM_V_ of a Voigt function can be reasonably approximated either by (Olivero & Longbothum, 1977 ▸) (Fig. 5 ▸)

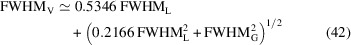

or by a polynomial of fifth order (Thompson *et al.*, 1987 ▸):

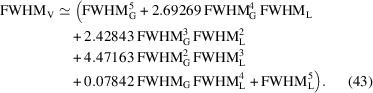

The integral breadth of a Voigt function is given as (Schoening, 1965 ▸)

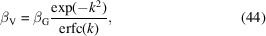

with the characteristic breadth ratio of a Voigtian *k* given by

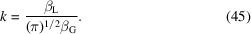

The FT of the Voigt function can be written as (Fig. 3 ▸, right)

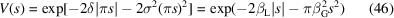

with an FWHM of

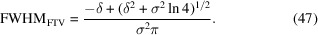

The convolution of two Voigt functions again is a Voigt function, a property that is used, for example, in the double-Voigt approach where two Voigtians are convoluted, each having its own Gaussian and Lorentzian fractions (Balzar, 1999 ▸).

For a long time, the exact computation of a Voigt profile was computationally expensive. One way to approximate a given Voigt function is to use a linear combination of a Gaussian and a Lorentzian, called the pseudo-Voigt approximation:

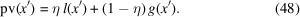

The mixing parameter 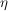 can be calculated using equation (43) in the range 0 ≤ η ≤ 1 by a cubic polynomial leading to the Thompson–Cox–Hastings (TCH) pseudo-Voigt function (Thompson *et al.*, 1987 ▸):

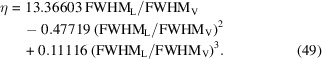

If a purely empirical approximation of a Voigt function is needed (as is generally sufficient for the determination of the IRF), a simplified version of the pseudo-Voigt function can be used:

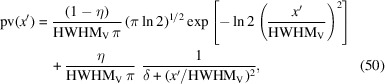

with its FT

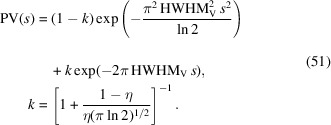




**Figure 6:**
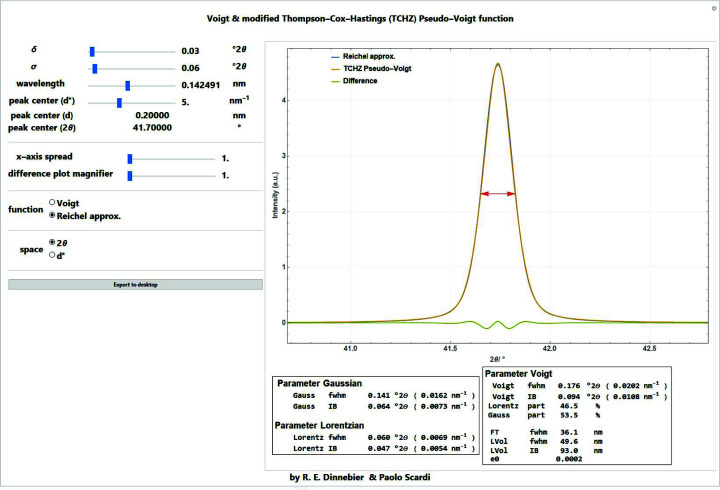
Screenshot of a *Mathematica* script for comparing the pure Voigt profile, the Reichel approximation and the TCH pseudo-Voigt profile for a given Gaussian standard deviation and Lorentzian HWHM in 2 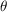 or 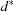 space. Values for crystallite size and microstrain were calculated using the formulas by Balzar (1999 ▸) under the assumption that the entire width of the peak is caused by either one. A good explanation of how size and strain values are calculated from the FWHM or the integral breadth of a Voigt function in the *TOPAS* (Coelho, 2018 ▸) software is given by Evans (2021 ▸).

**Figure 7:**
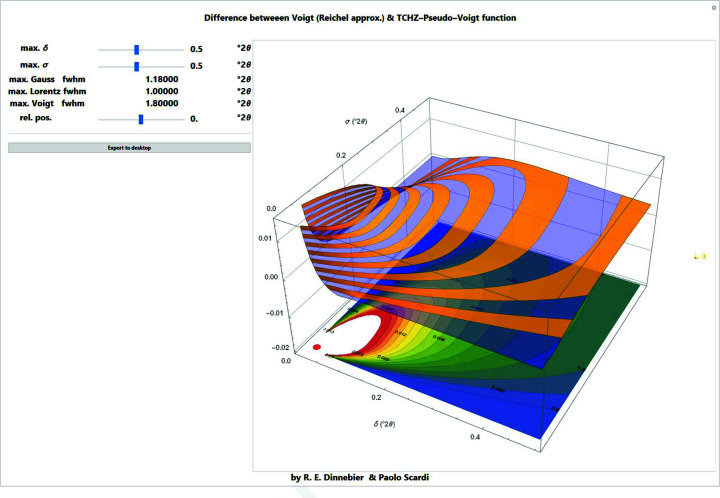
Screenshot of a *Mathematica* script for comparing the Reichel approximation of the Voigt function with the TCH pseudo-Voigt profile as a function of Gaussian standard deviation and Lorentzian HWHM at a given position relative to the peak centre in three dimensions.

Among the many approximations of the Voigt function found in the literature, the one by Chiarella & Reichel (1968 ▸), which was later modified by Abrarov *et al.* (2012 ▸), is a good compromise regarding speed and accuracy (Figs. 6 ▸ and 7 ▸):

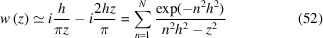

with 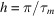 , and the chosen parameters τ_*m*_ and *N*, which determine the degree of approximation, typically 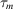 = 12 and *N* = 23. This function can be efficiently compiled in *Mathematica* (*Mathematica* stackexchange question 19884, https://mathematica.stackexchange.com/questions/19884/compiling-the-voigtdistribution-pdf), increasing the speed of computation considerably. Since the difference between a Voigt profile and the Chiarella and Reichel approximation is negligible (Fig. 7 ▸), the latter is used throughout the *Mathematica* scripts if a Voigt profile is required.

**Figure 8:**
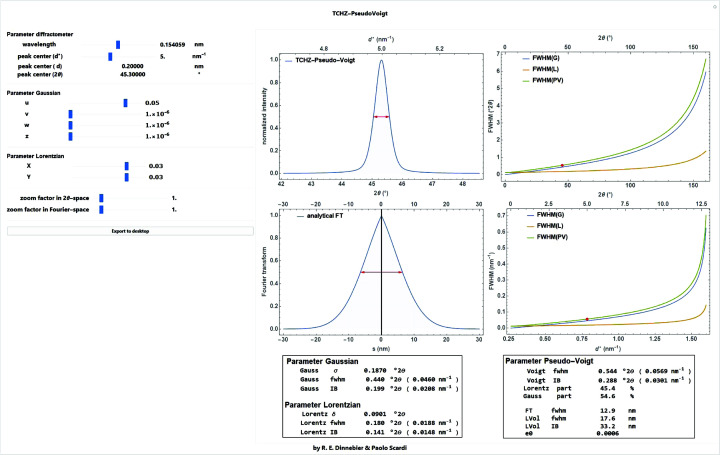
Screenshot of a *Mathematica* script for visualization of the dependence of the TCH pseudo-Voigt function and its Fourier transform on the diffraction angle. Values for crystallite size and microstrain were calculated using the formulas by Balzar (1999 ▸) under the assumption that the entire width of the peak is caused by either one.

The dependence of the Gaussian FWHM_G_ and Lorentzian FWHM_L_ on the 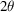 diffraction angle is usually described by low-order polynomials as a function of 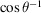 and 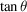 . While the 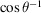 dependence follows directly from the Scherrer equation (Scherrer, 1918 ▸) and is attributed to the size of coherently scattering domains (often denoted as crystallite size), the 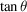 dependence is a measure of microstrain and can be derived from the full derivative of the Bragg equation (Bragg & Bragg, 1913 ▸). In an empirical but flexible way, the Gaussian FWHM_G_ and Lorentzian FWHM_L_ are often defined as (Thompson *et al.*, 1987 ▸; Young, 1993 ▸)

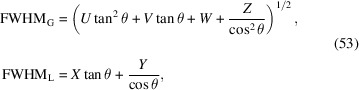

where *U*, *V*, *W*, *X*, *Y*, *Z* are refinable parameters. Apparently, the parameters have been chosen in such a way that *U* and *X* are somehow related to microstrain while *Z* and *Y* are kind of related to domain size (Von Dreele, 2008 ▸) (Fig. 8 ▸). Refining all parameters simultaneously generally leads to over-parameterization and high correlation, destabilizing the optimization process.

**Figure 9:**
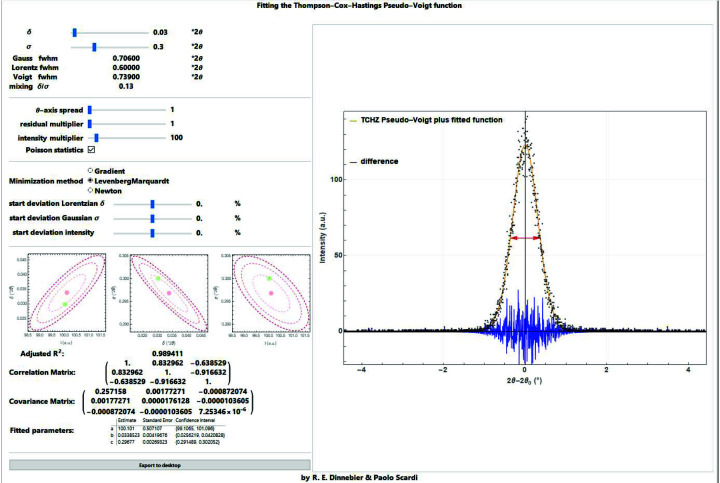
Screenshot of a *Mathematica* script for visualization of least-squares fitting using different algorithms of the TCH pseudo-Voigt perturbed by Poisson noise.

In order to retrieve the width parameters of a Voigt function which is blurred by Poisson noise, a variety of least-squares algorithms (*e.g.* gradient, Levenberg–Marquardt, Newton) are available (Press *et al.*, 2007 ▸). A *Mathematica* script has been written to evaluate the reliability of parameter retrieval depending on the amount of noise and to determine the degree of correlation between the fitting parameters (Fig. 9 ▸). The script is a useful tool to test and visualize data quality and the effect of noise on the refined parameters, applying different algorithms of minimization. It also introduces the concept of estimated standard deviation.

## 4. Instrumental aberrations to the peak profile

Only in a few cases can the IRF be fully described using a symmetrical Voigt-like profile function. In particular, the curvature of the Debye–Scherrer rings introduces a certain amount of asymmetry due to axial divergence if a rectangular receiving slit or silicon strip is cutting out only a small part of the ring. Each element in the optical pathway of the incident and diffracted beam convolutes its characteristic shape into the peak profile. The functions to describe the effects are either highly sophisticated based on physics and geometry, purely empirical, or a mixture of both. They are convoluted in real space, or multiplied in Fourier space, into the existing peak profile. In the following, a selection of four simple aberration functions in powder diffraction, which have proven to be useful, are discussed. Although this section is restricted to instrumental aberrations, the asymmetry introduced by the transparency effect, which depends on the preparation technique, is included since it is not related to the MS of the sample. Note that all functions presented here can be found in the *TOPAS* software (Coelho, 2018 ▸).

### 4.1. The box (hat) function

**Figure 10:**
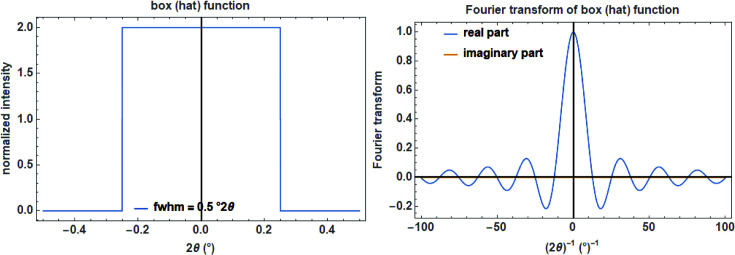
The box function on a 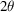 scale with a width of 0.5° 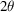 (left) and its real Fourier transform (right).

Several instrumental aberrations in the equatorial plane of a diffractometer are commonly described by a box (sometimes called top-hat) function. These include the width of the source, the thickness of the sample surface as projected onto the equatorial plane, the width of the receiving slit, the width of strips in position-sensitive strip detectors *etc*. The normalized (area under its graph is 1) box function with width *a* is defined as (Fig. 10 ▸, left)

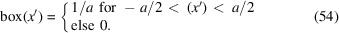

In the extreme case of *a* → 0, the box function turns into a δ function. For practical reasons this is achieved by setting *a* to a value <10^−5^.

The normalized (maximum value is 1) FT of a box function with the reciprocal variable *s* is a real function calculated as (Fig. 10 ▸, right)

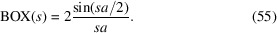

The convolution of a box function with itself leads to the triangular function. If the convolution process is repeated, the convoluted function approaches a Gaussian. In practice, this is realized after five or more convolutions.

**Figure 11:**
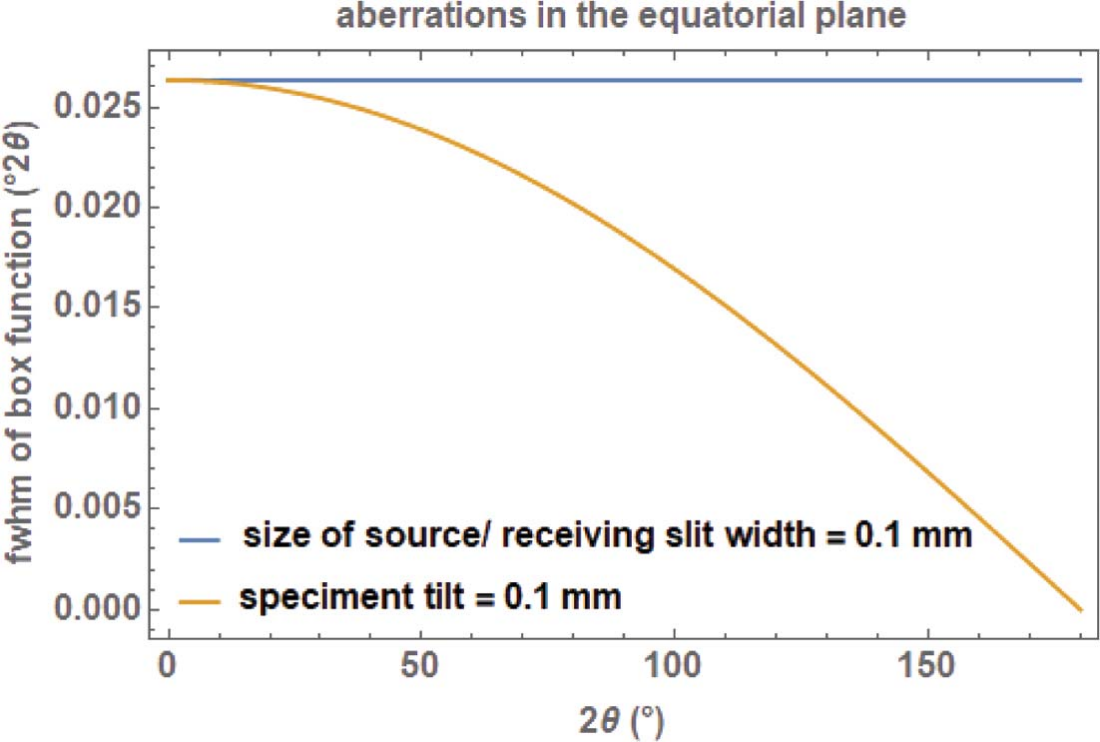
Aberrations in the equatorial plane of a diffractometer, assuming a secondary radius of 217.5 mm and a slit width (either receiving slit or size of the source) and a specimen tilt of 0.1 mm, as a function of diffraction angle 2θ.

To mimic the transmittance of a rectangular slit or a source with a width of *c* in mm (typically on the order of 0.1 mm) in the equatorial plane of a diffractometer with a secondary radius *R*_s_ in mm, a constant function for calculating the FWHM *a* of the box function is used (Fig. 11 ▸):

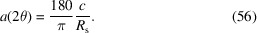

In order to calculate the width (FWHM) of the box function in degrees for the specimen tilt, the following function is used (Fig. 11 ▸):

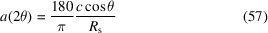

where *c* (in mm) represents the ‘thickness’ of the sample surface as projected onto the equatorial plane (Cheary & Coelho, 1992 ▸).

**Figure 12:**
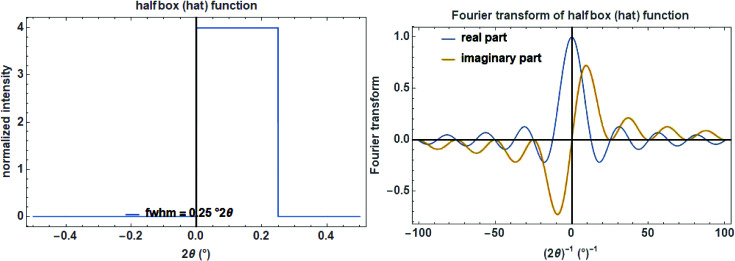
The halfbox function on a 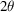 scale with a width of 0.25° 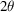 (left) and its complex Fourier transform (right).

In the case that an equatorial aberration is non-symmetrical (as for tube tails which might have different lengths on each side), a normalized halfbox function acting either on the left or the right side of a diffraction peak can be defined as (Fig. 12 ▸, left)

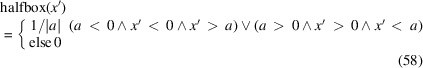

with its normalized complex FT (Fig. 12 ▸, right)

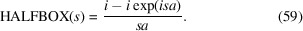

**Figure 13:**
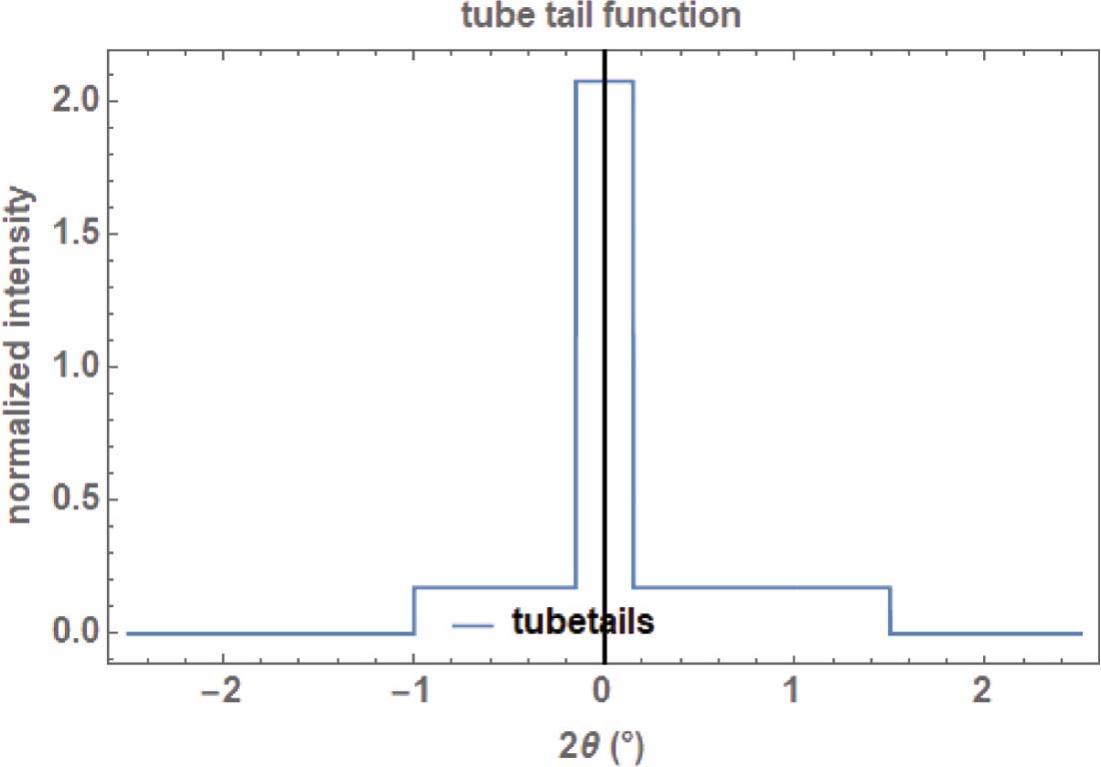
Tube tail function as a combination of a box function and two (left, right) halfbox functions for the tails.

To mimic the aberration caused by tube tails (Bergmann *et al.*, 2000 ▸), a combination of a box function describing the tube filament width in mm and two halfbox functions with an effective width of tube tails in the equatorial plane perpendicular to the X-ray beam in the negative and positive *z* direction in mm can be used. An additional parameter defining the fractional height of the tube tails relative to the main beam is then needed (Fig. 13 ▸).

**Figure 14:**
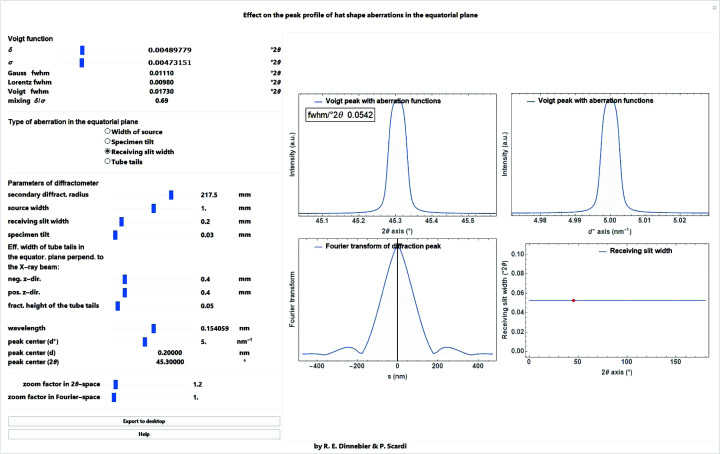
Screenshot of a *Mathematica* script dealing with the convolution of the box function into a Voigt profile. Different kinds of aberrations in the equatorial plane of a diffractometer assuming a secondary radius of 217.5 mm (originating from a receiving slit, the size of the source or the specimen tilt) as a function of diffraction angle 2θ can be selected.

A screenshot of a *Mathematica* script where the box/halfbox function from different kinds of aberrations in the equatorial plane of the diffractometer is convoluted into a Voigt profile is shown in Fig. 14 ▸.

### 4.2. The circles function

**Figure 15:**
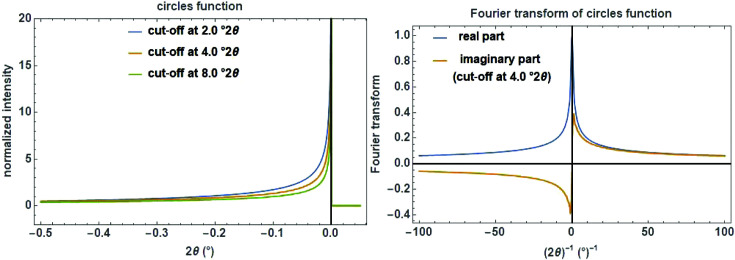
The circles function on a 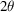 scale with three cut-off values of 2, 4 and 8° 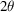 (left) and the Fourier transform of the circles function with a cut-off value of 4° 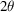 showing the real and the imaginary part (right).

A simple approximate function for modelling the asymmetry of a Bragg reflection is the circles function, with the cut-off value 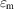 determining the curvature as an adjustable parameter (Fig. 15 ▸, left):

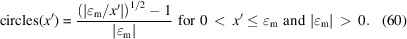

One of the main applications of this function is the phenomenological modelling of the peak asymmetry caused by axial divergence. This is predominantly due to the increasing curvature of the Debye–Scherrer rings at very low and extremely high angles which are cut by (typically) rectangular receiving slits of finite width (Cheary & Coelho, 1998 ▸). The complex FT of the circles function (Fig. 15 ▸, right) is defined as

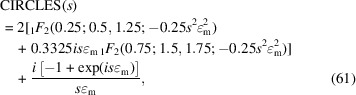

with the hypergeometricPFQ function 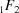 .

**Figure 16:**
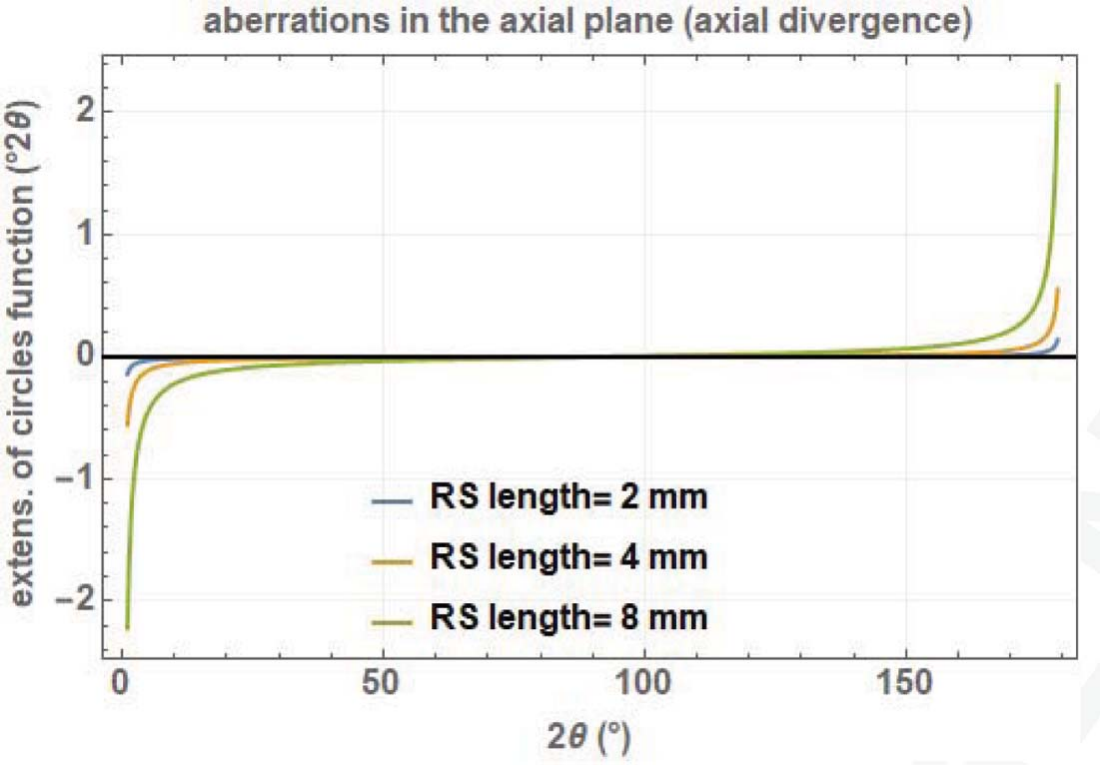
Cut-off values (= curvature) of the circles function to describe asymmetry due to axial divergence as a function of scattering angle for a diffractometer with a secondary radius of 217.5 mm and different receiving slit lengths in mm.

In order to model axial divergence with the circles function for angular dispersive data, the angular dependence of the cut-off value 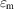 is well described by a tan (2θ)^−1^ type of function of the receiving slit length *c* in mm. Note that the asymmetry is reversed above 90° 2θ [actually closer to 120° 2θ as described by Cheary & Coelho (1998 ▸)] (Fig. 16 ▸):

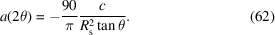

More sophisticated functions to describe the asymmetry due to axial divergence of divergent beam diffractometers can be found in the literature. The mathematical formalism to describe peak asymmetry due to the finite size of the detector receiving slit and the curvature of the Debye–Scherrer rings for parallel beam geometry has been developed by van Laar & Yelon (1984 ▸) and was implemented by Finger *et al.* (1994 ▸). Extensions to cover divergent beam geometry, common for laboratory diffractometers, have also been developed (*e.g.* Cheary & Coelho, 1998 ▸; Mendenhall *et al.*, 2017 ▸).

**Figure 17:**
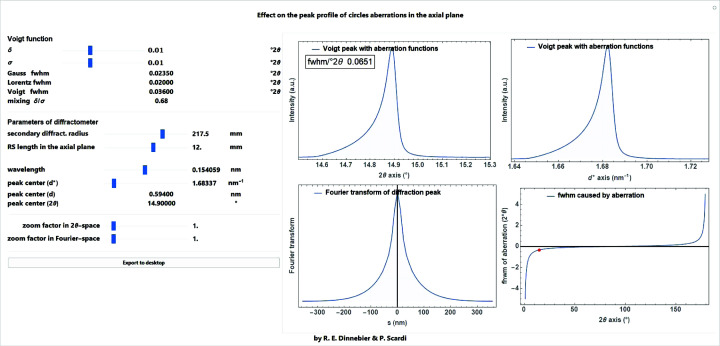
Screenshot of a *Mathematica* script dealing with the convolution of the circles function for modelling asymmetry due to axial divergence caused by the length of a rectangular receiving slit.

A screenshot of a *Mathematica* script where the circles function for describing asymmetry due to axial divergence of the diffractometer is convoluted into a Voigt function is shown in Fig. 17 ▸.

### 4.3. 1/*x* function

**Figure 18:**
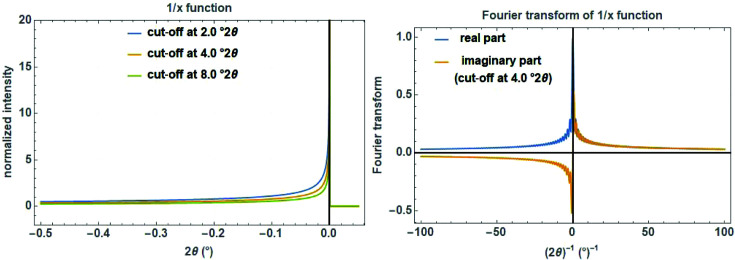
The 1/*x* function on a 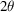 scale with three cut-off values of 2, 4 and 8° 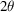 (left) and its Fourier transform with a cut-off value of 4° 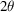 showing the real and the imaginary part (right).

Another aberration function to describe certain types of asymmetry of a Bragg reflection is the 1/*x* decay function, defined as (Fig. 18 ▸, left)

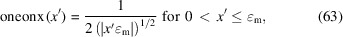

where the parameter 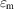 , which can be either positive or negative, is the cut-off value defining the extension of the asymmetric tail on the given length scale. The normalized complex FT of a 1/*x* function with the reciprocal variable *s* is calculated as (Fig. 18 ▸, right)

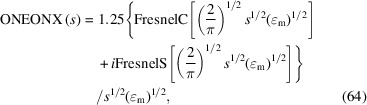

with the two Fresnel integrals

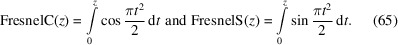

The 1/*x* function is mainly used for the phenomenological description of the equatorial divergence caused by a divergence slit (either fixed or variable) on the peak profile for angular dispersive data.

**Figure 19:**
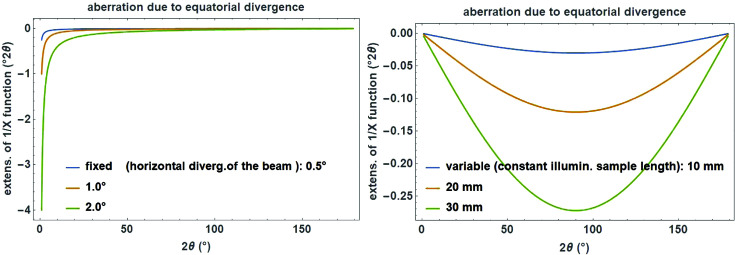
Angular dependence of the 1/*x* correction function for modelling the peak asymmetry due to equatorial divergence caused by a fixed divergence slit of 0.5, 1.0 or 2.0° opening (left) and a variable divergence slit with a constant illuminated sample length of 10, 20 or 30 mm (right). The secondary diffractometer radius is set to 217.5 mm.

The cut-off value 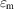 as a measure for the peak asymmetry is calculated for a fixed or variable divergence slit as (Cheary *et al.*, 2004 ▸)

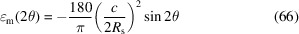

with the illuminated sample length *c* (in mm), which is constant if the divergence slit is variable but depends on the opening angle α (in degrees) if the divergence slit is fixed (Fig. 19 ▸):

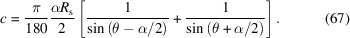

**Figure 20:**
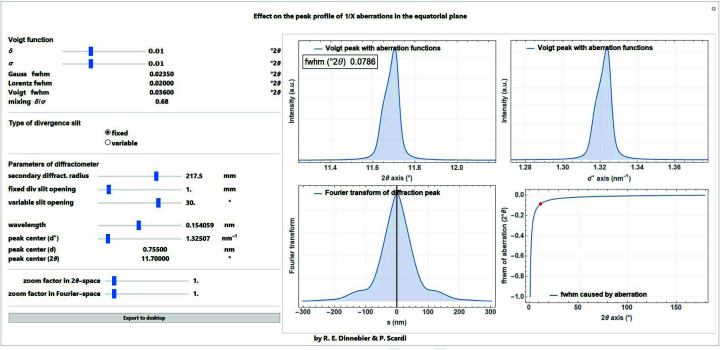
Screenshot of a *Mathematica* script dealing with the convolution of the 1/*x* correction function for modelling the peak asymmetry due to equatorial divergence caused by fixed or variable divergence slits.

A screenshot of a *Mathematica* script where the 1/*x* function for describing asymmetry due to fixed or variable divergence slits of the diffractometer is convoluted into a Voigt function is shown in Fig. 20 ▸.

### 4.4. The exponential function

**Figure 21:**
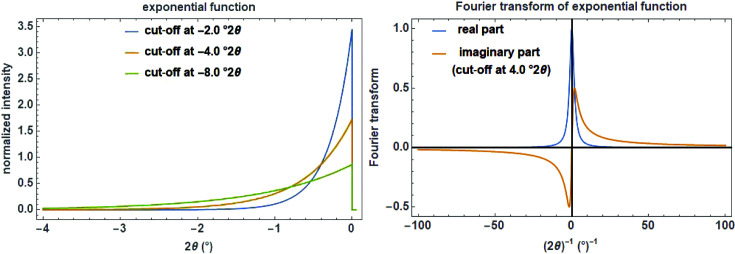
The exponential function on a 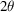 scale with three cut-off values of 2, 4 and 8° 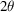 (left) and its Fourier transform with a cut-off value of 4° 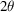 showing the real and the imaginary part (right).

Asymmetry of Bragg reflections can also be described by a normalized exponential asymmetry decay function of the type (Fig. 21 ▸, left)

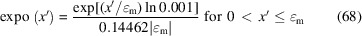

[ln(0.001) is a scaling factor which ensures that the value at 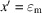 is 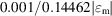 ] that is convoluted into the peak profile. The parameter 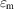 , which can be either positive or negative, is the cut-off value which defines the extension of the asymmetric tail on the given length scale and is a measure for the degree of asymmetry. The normalized complex FT of the expo function with the reciprocal variable *s* is calculated as (Fig. 21 ▸, right)

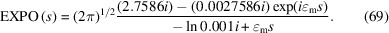




The exponential function can be useful for describing, for example, the highly asymmetric instrumental peak shape of TOF data or the effects of transparency on the peak shape in Bragg–Brentano geometry.

The main application of the exponential aberration function is to describe the peak asymmetry caused by the transparency effect, where a low-absorbing sample is filled in a deep cavity of a flat-plate sample holder in Bragg–Brentano geometry. The diffracted peak thus is a convolution from multi-diffraction at different depths with increasing absorption.

**Figure 22:**
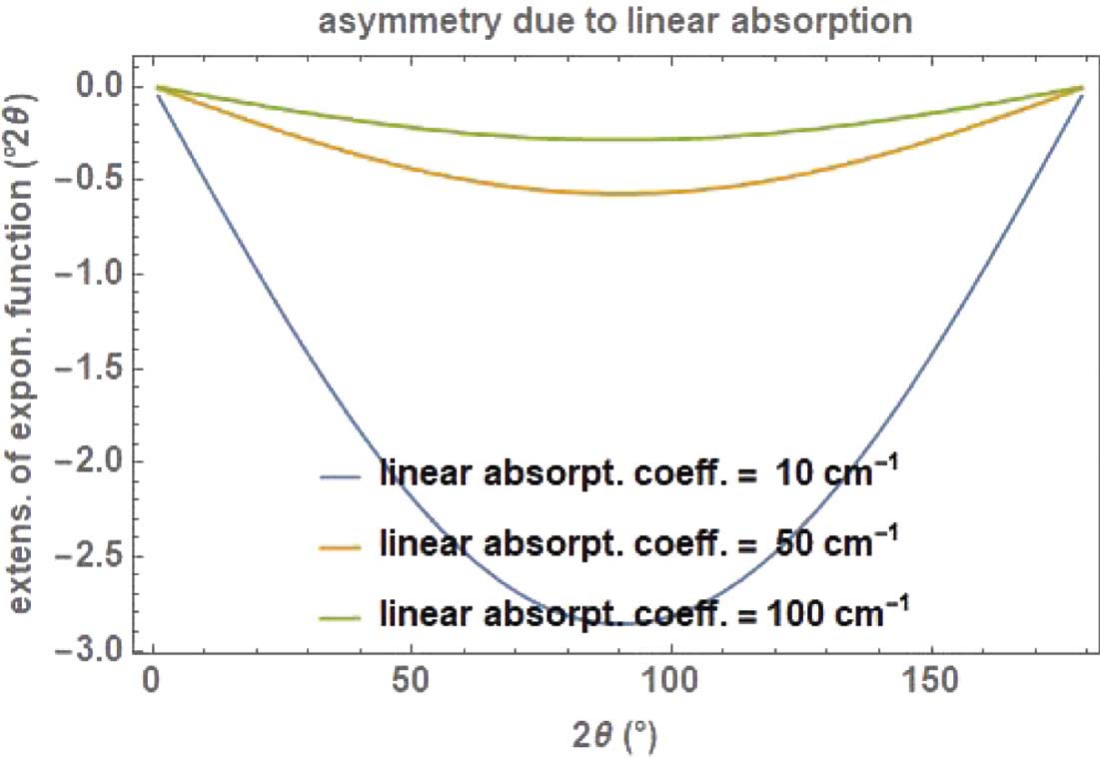
Angular dependence of the exponential function for modelling the peak asymmetry due to absorption for different absorption coefficients of 10, 50 and 100 cm^−1^. The secondary diffractometer radius is set to 217.5 mm.

The cut-off value 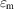 as a measure for the peak asymmetry is calculated for a linear absorption coefficient *a* in cm^−1^ (Fig. 22 ▸) as

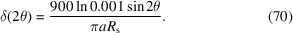

**Figure 23:**
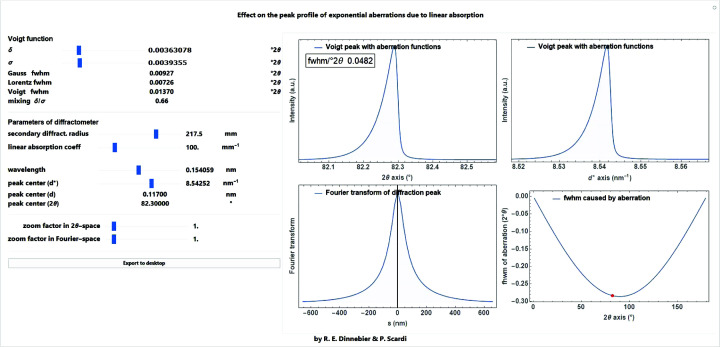
Screenshot of a *Mathematica* script dealing with the convolution of the exponential correction function for modelling the peak asymmetry due to the transparency effect caused by low linear absorption in flat-plate sample holders with sample-filled deep cavities.

A screenshot of a *Mathematica* script where the exponential function for describing asymmetry due to transparency is convoluted into a Voigt function is shown in Fig. 23 ▸.

## 5. Microstructural contribution to line profiles

Once determined, the instrumental contribution to the diffraction profile can be used constantly for a given instrument and setup. Whether it is modelled on the basis of fundamental parameters or with empirical approaches, the instrumental profile can be acquired experimentally, using appropriate powder standards (Cline *et al.*, 2013 ▸). Separately, the MS contribution varies for each case study and, therefore, there cannot be a universally valid model. This explains the practical use, described before, of empirical profile functions with adaptive parameters, justified by the fact that they ‘work well’, *i.e.* they fit the patterns in most cases. However, the interpretation of these parameters can be misleading due to the substantial arbitrariness in the choice of profile functions and their combination (Scardi *et al.*, 2004 ▸; Scardi, 2020 ▸).

The WPPM provides a description of the MS component of the profile based on a convolution of terms referring to physical parameters. The two normally prevailing effects – size and strain broadening – are introduced below, whereas other effects and more extensive discussions are reported in the literature (Scardi *et al.*, 2018 ▸; Scardi, 2020 ▸, and references therein). This choice corresponds to writing equation (6) as

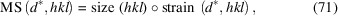

where the size is expressed as a function of lattice direction and strain as a function of both lattice direction and 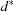 . This highlights an important property: when the diffraction profile is represented in reciprocal space, the size component, unlike the strain component, is independent of 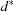 , which allows for separation of the two effects, *e.g.* through the classic Warren & Averbach method (Warren & Averbach, 1950 ▸; Warren, 1990 ▸). Both components, however, can depend on Miller indices (*hkl*), respectively, for the shape of the crystalline domains, and for the anisotropy of defect strain field and of the elastic medium. Note that, for small nanoparticles/domains, the separation of domain size and strain parameters becomes increasingly difficult, and it becomes ill-defined for very small or highly disordered domains in a crystallographic sense.

**Figure 24:**
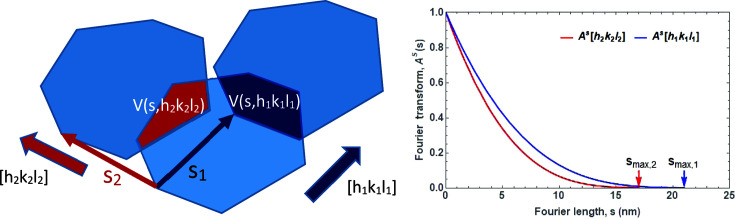
Geometrical interpretation of the Fourier transform of the size-effect profile component. The common volume function, *V*(*s*, *hkl*), along two different directions, [*h*_1_ *k*_1_ *l*_1_] and [*h*_2_ *k*_2_ *l*_2_] (left), with corresponding Fourier transforms (right); *s*_max_ stands for the maximum dimension in the crystalline domain along the given direction.

Within the limits of the tangent plane approximation (TPA) (von Laue, 1926 ▸; Beyerlein *et al.*, 2011 ▸), it can be shown that the FT of the profile component of the size effect, *A* ^S^(*s*), has a simple geometric interpretation (Wilson, 1962 ▸) (Fig. 24 ▸). In fact, it corresponds to the volume of intersection *V*(*s*, *hkl*) between the crystalline domain and the same domain translated by a distance *s* along [*hkl*], normalized to the domain volume, 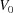 ,

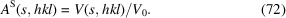




**Figure 25:**
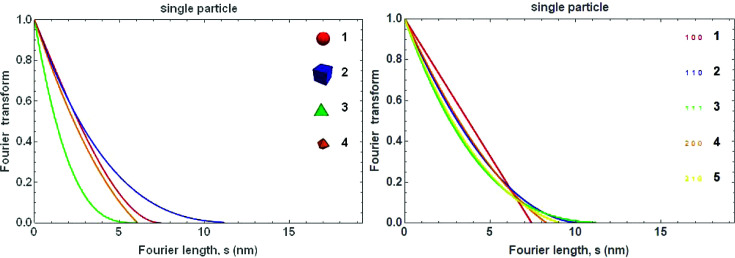
Fourier transform of the line profile given by spherical (1), cubic (2), tetrahedral (3) and octahedral (3) shaped particles in the [111] direction (left) and by cubic shaped particles for different [*hkl*] (right) (7.4 nm diameter for sphere, and edge for cube, tetrahedron and octahedron).

Simple geometrical shapes allow for an easy evaluation of *V*(*s*, *hkl*) in closed analytical form (Scardi & Leoni, 2001 ▸). Such is the case for frequently observed nanocrystal shapes, like the cube, tetrahedron, octahedron and sphere. The last is frequently assumed, even if only approximately valid, when nanocrystals do not have a specific shape but are reasonably equiaxed. Then the FT of the line profile given by spherical domains of diameter *D* is (Fig. 25 ▸, left, red curve)

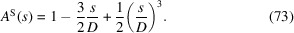

Apart from the spherical shape, *A* ^S^ is generally a function of *hkl*, a dependence requiring the choice of lattice orientation within the crystalline domain. Even if complex shapes and low-symmetry structures require numerical calculation methods, the calculation of *A* ^S^ can lead back to a tractable geometry problem (Leonardi *et al.*, 2012 ▸) (Fig. 25 ▸, right).

**Figure 26:**
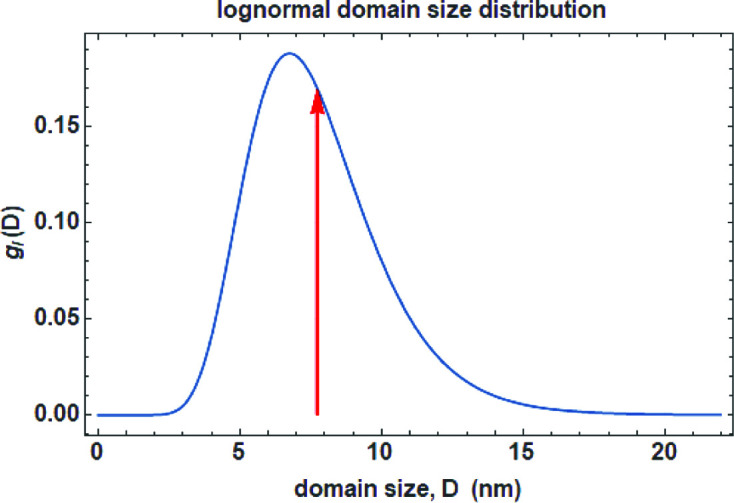
Lognormal distribution of sizes with lognormal mean μ = 1.95 and lognormal standard deviation 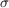 = 0.35, leading to a mean size of 7.47 nm (red arrow) and a standard deviation of s.d. = 2.68 nm.

It is unlikely that the domains, despite having the same shape, are of the same size. The problem can be addressed by considering a size distribution, as discussed by Scardi & Leoni (2001 ▸). A variety of distribution functions can be employed, but a lognormal distribution is often an appropriate choice (Fig. 26 ▸):

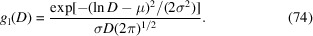

The lognormal mean (μ) and lognormal variance (σ^2^) give the central moments, 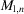 , where

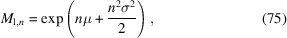

from which the mean and standard deviation, respectively, are

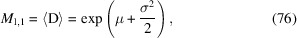




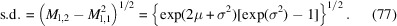

This distribution refers to a single dimensional parameter, *D*, the diameter of the sphere or edge of a cube, tetrahedron, octahedron *etc*. It is theoretically possible to use more distributions, for example, base diameter and height for a cylindrical shape, but the strong correlation between the different parameters (two for each distribution) makes the use impractical and refinement by least-squares usually unstable.

For a lognormal distribution of spheres, the FT 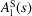 is (Scardi *et al.*, 2018 ▸)

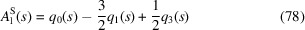

with

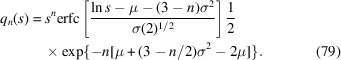

**Figure 27:**
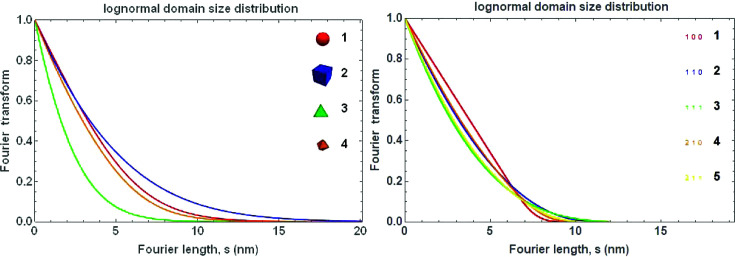
Fourier transforms of the line profile of a lognormal system of spherical (1), cubic (2), tetrahedral (3) and octahedral (3) shaped particles in the [111] direction (left) and of cubic shaped particles for different [*hkl*] (right). The parameters of Fig. 26 ▸ are used.

Analogous expressions hold for different shapes, which in this case involves a direction dependence, 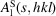 :

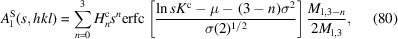

where the 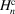 and 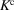 coefficients depend on the domain shape (*e.g.* 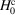 = 1, 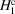 = −3/2, 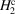 = 0, 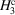 = 1/2, 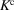 = 1, for spheres), and in particular 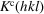 sets the maximum dimension in the crystalline domain along the given [*hkl*] (the formulas for the cubic crystal system require the condition *h* ≥ *k* ≥ *l*) [see Leonardi *et al.* (2012 ▸) for other shapes] (Fig. 27 ▸).

**Figure 28:**
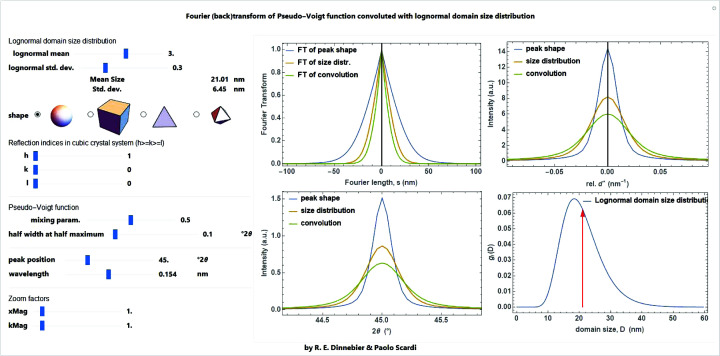
Screenshot of a *Mathematica* script dealing with the convolution of the size effect into a Voigt profile and its Fourier transform for a given lognormal distribution of a chosen shape.

The effect of a lognormal size distribution of particles with a given shape and size on the pseudo-Voigt profile and its FT is shown in Fig. 28 ▸.

The WPPM approach for the size effect is flexible enough and appropriate to deal with many real cases. It generally works if domains are not too small, because the TPA does not hold for very small sizes (∼5 nm is a realistic limit), and do not involve ‘non-crystallographic’ shapes like decahedra or icosahedra, which break translational symmetry.

The strain effect is more complex and varied than the size effect, and can be treated in a simple way only as a perturbation of otherwise perfect crystalline domains. In these terms, it can be shown that the FT of the strain component, 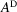 , of the line profile can be approximated by (Warren & Averbach, 1952 ▸)

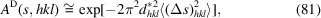

where 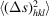 is the variance of the displacement distribution for any couple of scattering centres in the domain at a distance *s* along the direction [*hkl*]. This can also be written in terms of ‘microstrain’, defined as 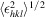 = 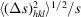 . Expressions for the microstrain can be derived for specific case studies, *e.g.* the presence of dislocations (Wilkens, 1970*a* ▸,*b* ▸), or by adopting phenomenological models (Adler & Houska, 1979 ▸). In both cases, basic features are (i) a marked dependence on the reciprocal-space vector [ 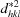 in equation (68)] and (ii) an anisotropy specific to the elastic properties of the material and of the type of lattice defects.

If one follows the Krivoglaz–Wilkens (KW) (Wilkens, 1970*a* ▸,*b* ▸) approach for dislocation strain broadening, the following expression results:

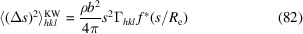

with ρ as the average dislocation density, *b* the Burgers vector modulus for the given slip system and *R*_e_ the effective outer cut-off radius of the dislocation strain field; f* is a known (Wilkens) function of *s*/*R*_e_ (Wilkens, 1970*a* ▸), and 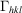 depends on the direction, in terms of a fourth-order invariant expression of the Miller indices. In the most general case (triclinic), this reads (Scardi *et al.*, 2018 ▸)

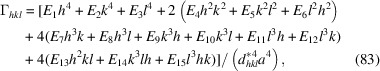

where *a* is the first unit-cell parameter according to the crystallographic conventions. Coefficients *E*_1_, *E*_2_,…, *E*_15_ can be calculated for specific strain fields, like that of dislocations, given the slip system and elastic constants (Martinez-Garcia *et al.*, 2009 ▸). The anisotropy factor, 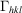 , for dislocation strain is also referred to as the average dislocation (or orientation) factor, 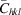 .

Symmetry reduces the number of terms in equation (83) (Scardi *et al.*, 2018 ▸) down to two for the most symmetric, cubic case, for which equation (83) simplifies to

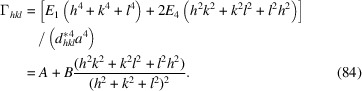




Values of *A* (= *E*_1_) and *B* [= 2(*E*_2_ − *E*_1_)] can be calculated (Martinez-Garcia *et al.*, 2009 ▸) but are also known in parametric form for a wide range of anisotropy (Zener) ratios and dislocation types in cubic systems (Ungár *et al.*, 1999 ▸; Dragomir & Ungár, 2002 ▸).

As already pointed out, it is also possible to use empirical expressions, such as that named after Popa–Adler–Houska (PAH), which extends the Adler & Houska (1979 ▸) approach by including the term of anisotropy introduced by Popa (Popa, 1998 ▸; Scardi *et al.*, 2015 ▸):

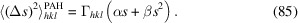

Formally, the anisotropy factor is the same as in equations (82) and (83), but in this case coefficients *E*_1_, *E*_2_, …, *E*_15_ become just fitting parameters, to be optimized together with α and β.

**Figure 29:**
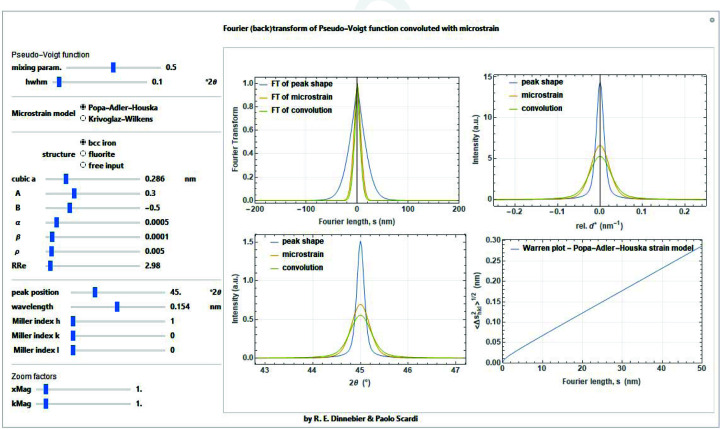
Fourier transform of the strain effect component of the line profile (top left), considering KW and PAH models, for the cases of ferritic iron (shown here) and of fluorite. Also shown are the corresponding line profiles, in reciprocal space (top right) and in 2θ space (bottom left), and Warren plot. See text for details.

Fig. 29 ▸ shows examples of applications of both models for two typical case studies, nanocrystalline body-centred-cubic (b.c.c.) iron and face-centred-cubic fluorite, respectively. It is interesting to note in these two examples, beyond the use of the two different models of equations (82) and (85), the opposite trend of the anisotropy factor. *B* in equation (84) is negative for b.c.c. iron (like most metals) and positive for fluorite (like many binary salts), which means that the stiff/soft directions are opposite: [*hhh*]/[*h*00] for iron and [*h*00]/[*hhh*] for fluorite. As a consequence, (*hhh*) line profiles in iron tend to be less broadened than the (*h*00) profiles, apart from the 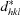 dependence, and vice versa for fluorite. Strain broadening anisotropy definitely helps the separation of size and strain effects.

MS effects can be visualized by two common representations, the Williamson–Hall plot (Williamson & Hall, 1953 ▸) and the Warren plot (Warren & Averbach, 1950 ▸). The former, consisting of a plot of the integral breadth (IB = peak area/peak maximum intensity) versus 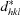 for as many peak profiles as are available in the experimental pattern, is also used for a preliminary assessment of the size and strain parameters. Note, however, that this analysis (also known as the Williamson–Hall method) is affected by the arbitrary choice of additivity for the size and strain IBs (Scardi *et al.*, 2004 ▸). On the basis of the WPPM approach, instead, we can calculate the IBs from the FTs, for individual profile components and their convolution:

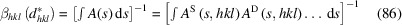

where the ellipsis indicates that other terms contributing to the profile can be added (*e.g.* the instrumental profile). The dependence on *hkl* is invariably present in the strain term, whereas it is absent in the size effect for the frequent case where a spherical domain shape (average) is adopted. IBs of individual profile components are also easily obtained (in analytical form in some cases) as

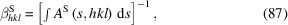




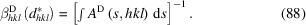

**Figure 30:**
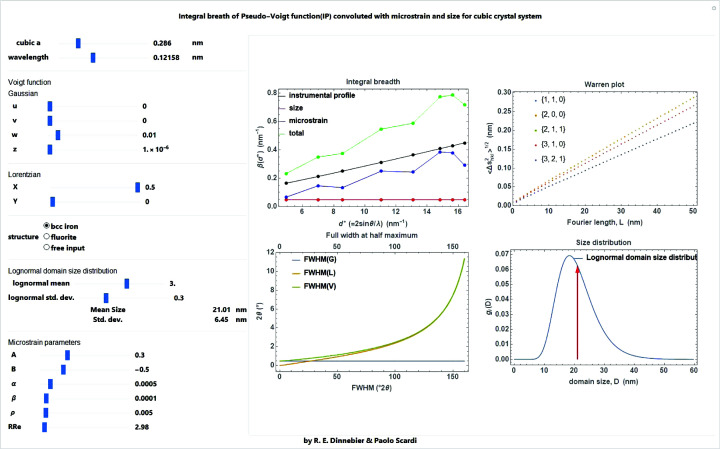
Integral breadth plot (or Williamson–Hall plot) (left) for the case of Fig. 29 ▸, considering contributions from instrument, domain size/shape and microstrain; corresponding Warren plot for some representative crystallographic directions (right).

Analogous expressions hold for other possible profile components. All integrations virtually extend from −∞ to +∞, but more realistically within a finite range, determined by the maximum length for a given domain shape and the dispersion of the size distribution. Fig. 30 ▸ shows examples of the Williamson–Hall plot where the different dependence on 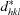 and on *hkl* can be seen. The different trends, with the constant size term and the characteristic anisotropy of the strain term, further demonstrate the possibility of separating the different effects.

The Warren plot concerns only the strain component of the profile and provides a convenient visualization of the dependence on *s*, the Fourier variable (a physical length in the crystalline domain), and anisotropy. It is a plot of 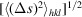 , the standard deviation of the displacement distribution, as a function of *s*, for different directions [*hkl*]. Examples are shown in Fig. 30 ▸.
